# Reading Eye Movements Performance on iPad vs Print Using a Visagraph

**DOI:** 10.16910/jemr.14.2.6

**Published:** 2021-09-14

**Authors:** Alicia Feis, Amanda Lallensack, Elizabeth Pallante, Melanie Nielsen, Nicole Demarco, Balamurali Vasudevan

**Affiliations:** Arizona College of Optometry, Midwestern University, USA

**Keywords:** Eye movement, saccades, reading, attention, Visagraph, iPad, print

## Abstract

This study investigated reading comprehension, reading speed, and the quality of eye movements while reading on an iPad, as compared to printed text. 31 visually-normal subjects were enrolled. Two of the passages were read from the Visagraph standardized text on iPad and Print. Eye movement characteristics and comprehension were evaluated. Mean (SD) fixation duration was significantly longer with the iPad at 270 ms (40) compared to the printed text (p=0.04) at 260 ms (40). Subjects’ mean reading rates were significantly lower on the iPad at 294 words per minute (wpm) than the printed text at 318 wpm (p=0.03). The mean (SD) overall reading duration was significantly (p=0.02) slower on the iPad that took 31 s (9.3) than the printed text at 28 s (8.0). Overall reading performance is lower with an iPad than printed text in normal individuals. These findings might be more consequential in children and adult slower readers when they read using iPads.

## Introduction

There is a significant increase in the use of electronic devices both for office work and personal needs by adults of different ages and also with different occupational needs. Children have also started using electronic devices like iPad for both educational as well as recreational needs. Their screen time has increased significantly since COVID-19 pandemic as classes were conducted online and electronic devices were the medium of education.

It has been reported that the use of electronic devices leads to to visual symptoms that may include headache, eyestrain, dry eye, diplopia, and blurred vision (21; 9; 22 ; 15). These have been reported at both near and/or when gazing into the distance after short duration (e.g., 20 minutes) use of electronic devices (4). This has been confirmed with the use of a kindle for as low as 12 minutes, compared to that of a hard copy (10). These symptoms can be more prevalent with the increased use of electronic displays, not just with college and graduate students, but with the increased use in middle and high school children as well. Portello et al. (15) measured the blink rate in normal subjects who read using a desktop computer for 15 minutes at 50 cms and reported that they had a blink rate that was inversely proportional to the symptom score. Both incomplete blinks and partial blinks increase the symptoms associated with the use of computers. In another investigation by the same lab, Chu et al. (4) reported that a higher number of incomplete blink rate was observed with the use of a computer device than a hard copy. These incomplete blinks that cause dry eye symptoms also adds to the rest of the visual symptoms with the use of display devices (10).

Reading is a complicated task that involves different eye movements such as fixations and saccadic eye movements (5 ). Saccades range in duration of 20-30 ms and span 7-9 letters, while fixations average 250-300 ms in duration (18). The length of the fixational pauses is related to the cognitive process that is responsible for the recognition of these words (17; 12).

There are different metrics to study eye movements in both normal and abnormal reading patters that includes: number of fixations, saccade length, regressions, return-sweep saccades, span of recognition, perceptual span, fixation duration, reading rate (3), etc., in addition to the understanding of the role of visual content (14) and comprehension levels (13; 23), both of which are necessary for successful understanding of the text material. There is a difference in the reading comfort between an e-ink based devices vs LCD/LED based devices such as iPads. On the contrary, these devices, in general, provide adequate brightness levels when set to the users need that makes it easier to read under reduced external illumination. While both print-based and electronic display-based media have their own merits and demerits, it turns out to be a user preference as to which mode works best for an individual for a given task as compared to the other. A study by Bababekova et al. (1) that investigated the font size and viewing distance of handheld smart phones determined that the mean working distances were shorter (26-40 cms) with the digital devices than the hard copy paper. These findings have implications when treating patients especially younger children with asthenopia that arises with the use of these handheld devices.

Comparisons between electronic devices and print reading on fatigue levels have been reported in the literature (e.g., 7). Many of these studies have reported on the reading speed, comprehension, accuracy, and only a few of them use an eye tracking methodology (e.g., 20; 2). Many physical characteristics have an influence on reading online such as font size, screen dimensions, contrast and luminance, and line length. Siegenthaler et al. (20) compared the effect of fatigue and visual strain on e-Ink vs backlit LCD on 10 subjects for an extended period of reading of 70 minutes. They reported that there was no significant difference between either of these devices. Benedetto et al. (2) compared the effect of prolonged reading on visual fatigue using three different mediums (LCD, e-ink and paper). They reported that with a prolonged reading duration of approximately 70 minutes for each session (performed on a separate day), reading with LCD triggered more visual fatigue than e-ink, in addition to the decrease in the number of blinks. Reading with printed text had the highest subjective preference followed by e-ink and then LCD.

Few studies have been performed to study the eye movements along with the subjective preference of reading. For instance, Kretzschmar et al. (11) measured the eye movements on thirty-six young and twenty-one elderly adults who read short texts on three different reading devices: a print page, an e-reader and a tablet computer. They used a video oculographic technique that involved using 2 video cameras placed below the computer screen, while reading from the iPad, eReader and printed book. Their eye movements and electroencephalogram (EEG) was recorded, they answered comprehension questions after reading. They reported that the mean fixation duration was different only when reading from the computer display, and for the other 3 conditions, there was no significant difference. The percentage of regressions with respect to the total amount of fixations was comparable between eReading devices and the printed book. The percent of regressions over total number of fixations was not different between the medium. Subjects preferred the print over the two electronic devices as their preferred reading medium.

Studies investigating objective eye movements characteristics and subjective feedback are very limited. Hence, the aim of the present study was to measure the eye movement characteristics, and subjective preferences for an electronic display like iPad vs printed text during a very short reading duration task in young individuals.

## Methods

### Participants

Thirty-one subjects were recruited for this cross-sectional study. Initial pilot study was performed on 5 subjects that provided the mean and SD of the reading rate (WPM) needed to calculate the sample size. The print reading rate was 339 (98) and iPad reading rate was 279 (89) that produced a calculated effect size of 0.639. Sample size calculation was performed using G-power software and a two tailed paired test was performed using an alpha of 0.05 and power of 0.9, hence a sample size of 28 was determined for reading rate comparison. Subjects were primarily students from the college of optometry, with mean (SD) age of 26.2 (3.8) years. The study was conducted between June 2018 and January 2019. Subjects were enrolled in the study if they were in the ages of 20-40 years, non-presbyopic, best corrected near visual acuity of 20/20 in each eye. Any subject with strabismus, amblyopia and dyslexia were excluded from the study. Any subject with abnormal binocular vision as identified with abnormal NPA and NPC and vergence ranges were excluded from the study. The study followed the tenets of the Declaration of Helsinki and was approved by the Midwestern University IRB Office. All participants signed a written informed consent. All the subjects who were interested to volunteer and signed the consent form, completed the study. A campus wide email was sent to all the colleges within the university and subjects were recruited based on their interest to volunteer in the study.

### Materials

All the data were collected in a single visit. Subjects were given 2 different passages to read. These passages were presented on a hard copy as well as displayed on an iPad. Both mediums were placed at a fixed distance of 40 cms from the subject’s eyes. Lighting levels in the room measured with a light meter was ~50 lux. Both the iPad and paper were placed on the table and a constant working distance and room illumination of ~50 lux was maintained to avoid inducing any change between the 2 different devices. The order of presentation was randomized between an iPad vs printed text and the two different standardized passages from the Visagraph book. The paragraphs were each 12 lines, typed double-spaced using 12-point times bold font which is approximately 20/70 near Snellen equivalent (6). During each of the two reading modes, eye movements were recorded objectively using a Visagraph system (5). Visagraph is a low-cost portable eye-movement system that uses infrared light emitting goggles and sensors to measure corneal reflections at a sampling rate of 60 Hz (Taylor, 2009).

### Procedure

Visagraph provides eye-movement data pertaining to reading that is comparable to a more sophisticated eye movement recording system like Eyelink with regards to the key metrics reported in this study (19 ). Subjects were asked to fixate on the first word and when instructed to ‘start’, they read from the top left of the text to the bottom right and closed their eyes when completed. Prior to the commencement of the reading session, subjects were informed that there would be a comprehension test at completion of the reading passage with a need to obtain 70 percent score or higher to pass. Subjects were offered a trial session prior to the real measurements. Each of the two reading-sessions was for a very short duration. One of the Visagraph passages was electronically captured and used on an iPad to mimic the similar appearance to the print version by calculating the right size of the optotypes to avoid reducing any variability. Both the passages were from the College-grade level text passages. Ambient lighting levels were used and made approximately similar between the iPad and print version. Every effort was made and standardized to control for the glare and the size of the text between the two modes, thereby providing subjects a comparable reading interface. At the completion of each reading passage, the following metrics were captured by the visagraph software: fixations per 100 words, fixation per character, fixation duration, regressions/100 words and reading rate. Since it has been reported that the number of fixations increased with the character size in the text read, fixations per character was also studied (8).

Subjects had to take a survey both before and after the completion of the reading passages. Table 1 summarizes all the questions that were asked prior to the reading survey.

**Table 1.** Survey questions asked prior to the reading task.

Questions		
What do you tend to use more often when studying?	Hard Copy	Laptop, Phone, Tablet, Desktop
Do you prefer to buy a hard copy of your textbook or read it online?	Hard Copy	Digital Device
If you didn’t have to worry about price, would you prefer to buy a hard copy of your textbooks?	Yes	No
On which device do you feel you are a more efficient reader/studier?	Hard Copy	Digital Device
Which device do you think provided you with the highest reading speed and comprehension scores on today’s test?	Hard Copy	Digital Device

To compare each of the eye movement metrics obtained from the Visagraph, paired t-tests were performed between the findings of the iPad and print. Pearson correlation analysis was also performed to identify any possible associations. Scores from the survey were analyzed to study the trends. Statistical analysis was performed using SPSS (V25.0).

We hypothesize that there will be a better reading comprehension, reading speed, and overall better eye movement quality on paper devices when compared to a digital device.

## Results

A total of 31 subjects were enrolled in the study. The mean (SD) for the number of fixations per 100 words for print and iPad reading passages was 75.58 (19.73) and 79.58 (21.86), respectively. Paired t-tests revealed that the number of fixations were not significantly different (p=0.11). Similarly, the mean (SD) for the number of fixations per character for print and iPad reading passages was 0.13 (0.03) and 0.14 (0.03), respectively. Paired t-tests revealed that the number of fixations were not significantly different (p=0.12). The mean (SD) for the number of regressions per 100 words for print and iPad reading passages was 8.10 (2.28) and 10.23 (2.40), respectively. Paired t-tests revealed that the number of regressions per 100 words were not significantly different (p=0.15). The mean (SD) of the fixation duration for print and iPad reading passages was 260 (40) and 270 (40), respectively. Paired t-tests revealed that the fixation duration was significantly different (p=0.04). The mean (SD) for the number of reading rate for print and iPad reading passages was 318.61 (89.07) and 294.67 (89.66), respectively. Paired t-tests revealed that the reading rate was significantly different (p=0.03). The mean (SD) of comprehension for print and iPad reading passages was 95.16 (7.24) and 94.5 (8.09), respectively. Paired t-tests revealed that the comprehension rate was not significantly different (p=0.7). The mean (SD) of reading duration for print and iPad reading passages was 28.62 (8.07) and 31.39 (9.37), respectively. Paired t-tests revealed that the reading duration was significantly different (p=0.02). The mean (SD) of return saccades for print and iPad reading passages was 1.54 (0.35) and 1.55 (0.49), respectively. Paired t-tests revealed that the saccades return was not significantly different (p=0.83). See Table 2.

**Table 2.** Summary of mean and standard deviation of different eye movement metrics. Paired t-tests were performed, and its p-values are included. Significant p-values are marked with asterisk.

Metrics	Mean	Std. Deviation	p-value
print fixations/100 words	75.58	19.73	0.11
iPad fixations/100 words	79.58	21.86	
print regressions/100 words	8.10	2.28	0.15
iPad regressions/100 words	10.23	3.40	
Print fixation duration (ms)	260	40	0.04*
iPad fixation duration (ms)	270	40	
Print reading rate (wpm)	318.61	89.07	0.03*
iPad reading rate (wpm)	294.68	89.67	
Print grade level equivalent	12.12	1.91	0.08
iPad grade level equivalent	11.48	2.49	
Print correct comprehension	95.16	7.24	0.74
iPad correct comprehension	94.52	8.10	
Print time read (secs)	28.62	8.07	0.02*
iPad time read (secs)	31.39	9.37	
Print analysis reliability	84.13	16.95	0.75
iPad analysis reliability	82.97	19.19	
Print return saccades	1.54	0.35	0.83
iPad return saccades	1.55	0.49	

Survey scores reported that the top 25^th^ percentile of the subjects tend to use hard copy material more than the laptop, phone, tablet or desktop (Table 3). Interestingly, subjects also revealed that the top 25^th^ percentile will score more with the use of a hard copy than a digital device. This choice remained the same for the top 25^th^ percentile when they were asked for their choice of medium that they would prefer. And, it remained the same for the question-which device do you prefer using? The 50^th^ and 75^th^ percentile of the respondents read on their laptop more often. See Table 3 for a summary of the percentile scores.

**Table 3.** Summary of the percentile scores from the questionnaires.

**Percentiles**	**Tend to use more**	**What device do you think you will score better with?**	**Post-exam: most efficient?**	**What device do you prefer using?**
25^th^	Hard Copy	Hard Copy	Hard Copy	Hard Copy
50^th^	Laptop	Hard Copy	Hard Copy	Hard Copy
75^th^	Laptop	Hard Copy	Digital Device	Hard Copy

## Discussion

The primary findings of the current study include: fixation duration was longer with iPad compared to the printed text, and reading rate was slower with an iPad than print.

### Fixation Duration

Significant portions of time spent during a reading task is fixations. Fixation duration had been accepted as a metric to understand the cognitive aspect of reading. Findings from the current study indicate that the mean number of fixations between the two modes, i.e., iPad vs print were not significantly different, however the mean fixation duration was 260 ms (40) for print vs 270 ms (40) for iPad, and they were significantly different. Given the small difference in the mean values, it appears that the two modes are approximately similar in their fixation duration. The findings were comparable to the study by Siegenthaler et al. (20) who investigated the effect of fatigue and visual strain following reading on LCD vs e-ink displays, reported that the mean (SD) of the fixation duration was 205 ms (78) for e-ink and 204 ms (58) for LCD and they were not significantly different. Similarly, in another study, Zambarbieri and Carniglia (24) compared three different eReading tools, i.e., desktop PC, iPad and Kindle eReader with a printed book, and they reported that the mean (SD) of the fixation duration with iPad was 208 ms (92) and with a book was 215 ms (92). Fixation duration was not significantly different between the book and the iPad. These results are similar in trend to the current study, with slightly lower fixation durations due to the nature of the reading material and the shorter duration of the reading task.

### Reading speed, regressions, and comprehension

For most of the college-age readers, the reading speed varies based on the topic with science-based material producing 235 wpm vs a light fiction material producing 365 wpm (Rayner & Pollatsek, 1989). As expected, regressions were much higher in the difficult material at 17-18% vs 3% for light fiction material. While the current study involved optometry students, material offered to them was the equivalent of light material at the college grade level. The mean reading speed with this material was 318 wpm for print vs 294 wpm for iPad. The mean reading rate with iPad was slower than the print. These findings are in alignment with that of Hue et al. (10) who compared the reading rate in Kindle, iPad and hardcopy on 20 young subjects. They reported a mean reading rate of 190.4 with hard copy vs 95.9 with iPad. In the current study, the reading material was presented in a counterbalanced manner between individuals, with half the subjects reading on the iPad first and the rest reading the print. The sequence of presentation did not have an effect on the reading speed. Reading passage used in the iPad was a very high-quality scanned version of the college level reading passage from the Visagraph book. The level of difficulty between the two modes are very similar. Reading speed was found to be slower when using an iPad and there are few possible reasons for the same. The number of regressions was ~8% for the print vs ~10% for the iPad. Similar to the findings of Zambarbieri and Carniglia (24), regressions in the current study are not statistically significant. In addition, the comprehension scores between the two modes are not significantly different. The accommodative lag is defined by the difference between the measured accommodative response produced for a given accommodative stimulus and was calculated between the two different reading modes. The findings on accommodative lag (16) has been mixed and in general, the current study did not measure the accommodative lag. Increased visual fatigue could also be a reason. Benedetto, et al. (2) reported a higher visual fatigue with LCD screen, compared to an electronic ink and print copy. This visual fatigue might be causing the subject’s more regressive eye movements that might be the case of the lower reading rate in an iPad, that uses LCD technology. This lower reading rate could be the outcome needed to understand the reading material and maintain the needed comprehension scores.

### Questionnaires

Subjective feedback for the use and preference of a medium (print vs iPad) was assessed. Top 25 percentile of the subjects preferred to study from the hard copy than when using their iPad and they also believed that it would help them score better. These findings are similar to that of Benedetto et al. (2) who compared a Kindle Paperwhite and Kindle HD (that uses LCD, similar to an iPad) to a traditional textbook. Interestingly, Benedetto, et al. (2) used French language-based novel for the 3 different reading sessions with each of the device and employed a longer reading duration. Results from the current study with a much shorter reading duration are similar in trend. Every subject learns differently, and they use some sort of technological device like a laptop and/or iPad, in addition to printed material to study for the examination. Most of the subjects used different devices for different durations of time for their educational needs that they feel comfortable with. Similar sentiments were also shared by Kretzschmar et al. (11) who reported their findings on both younger and older cohorts of subjects.

### Future studies

While the current study has highlighted the slightly slower reading speed with an iPad, in comparison to print for a very short duration of time in young adults, future studies should address this with younger children who are getting started with the use of an iPad for their education needs. Studies should also be performed on children with reading difficulty to see if the use of an iPad is a comfortable alternate to that of the traditional print books as the use of electronic devices like iPad should not set them back further.

### Limitations of the study

Most of the studies reported in the literature have had a reading task of several minutes, the current study explored a very short reading task of approximately 1 min in duration. Reading duration was short as subjects were reading passages from the Visagraph that had only 12 lines of text with 10 words in each line in a passage and most subjects were able to read them in less than a minute. The study duration was preferred as it enabled us to see if there were differences in the reading eye movements on the effect of digital devices for short durations of reading. The present study did not assess subjectively for fatigue, only key eye movement parameters, and comprehension in young adults were assessed. While the findings from the present study might not be directly comparable with that of others, it provides an important addition to the existing literature providing the role of motor and cognitive aspects of reading with an iPad, which is the commonly used handheld device for higher grade digital media of education in several countries.

### Ethics and Conflict of Interest

The authors declare that the contents of the article are in agreement with the ethics described in http://biblio.unibe.ch/portale/elibrary/BOP/jemr/ethics.html and that there is no conflict of interest regarding the publication of this paper.

### Acknowledgements

None.
